# Draft Genome Sequences of Eight Enterohepatic *Helicobacter* Species Isolated from Both Laboratory and Wild Rodents

**DOI:** 10.1128/genomeA.01218-14

**Published:** 2014-11-26

**Authors:** Alexander Sheh, Zeli Shen, James G. Fox

**Affiliations:** a Division of Comparative Medicine, Massachusetts Institute of Technology, Cambridge, Massachusetts, USA; b Department of Biological Engineering, Massachusetts Institute of Technology, Cambridge, Massachusetts, USA

## Abstract

The draft genome sequences of eight enterohepatic *Helicobacter* species, *H. muridarum, H. trogontum, H. typhlonius*, and five unnamed helicobacters, are presented here. Using laboratory mice pervasively infected with helicobacters, we characterized the presence of known virulence factors.

## GENOME ANNOUNCEMENT

Enterohepatic *Helicobacter* species (EHS) are Gram-negative, microaerophilic, spiral-shaped bacteria that colonize the mucosa of the gastrointestinal tract and/or the livers of mammals, including humans, and birds (1, 2). Natural enterohepatic *Helicobacter* sp. infection is prevalent in 88% of research mouse colonies worldwide (3). Our previous work reported the high prevalence of *Helicobacter hepaticus, Helicobacter rodentium, Helicobacter bilis*, and *Helicobacter typhlonius* in research mouse facilities (3). Previously, we have sequenced multiple EHS, including *H. bilis, Helicobacter pullorum, H. hepaticus*, *Helicobacter cinaedi*, and *Helicobacter canadensis* (4, 5). While most infected mice develop minimal pathological changes, susceptible strains exhibit typhlocolitis and hepatitis, which can progress to colon cancer and hepatocellular carcinoma (6). Previous studies have shown that *Helicobacter* infections can affect experimental outcomes in cancer studies and confound study results (7–9).

Furthermore, studies have highlighted the potential zoonotic nature of EHS species, as EHS isolated in rodents or birds, such as *H. cinaedi, H. canadensis, H. bilis*, and *H. pullorum*, have been identified in patients with diarrhea, cholecystitis, and biliary neoplasia (10–12), and it is well-documented that EHS can also infect other animal species, such as dogs, cats, geese, rhesus macaques, hamsters, gerbils, guinea fowl, and chickens (13–31).

In this report, we announce the whole-genome sequencing of eight EHS, including *Helicobacter muridarum* ST1, *Helicobacter trogontum*, *H. typhlonius*, as well as unnamed *Helicobacter* species (Massachusetts Institute of Technology [MIT] strains 01-6451, 03-1614, 03-1616, 05-5293, and 11-5569). These isolates were obtained from cecal, colon, and fecal samples of either laboratory or wild mice and rats. The isolates were sequenced using Illumina MiSeq sequencing technology, as described previously (32). The 250-bp paired-end sequencing reads generated by MiSeq were assembled into contigs using Velvet (33). The sequences were annotated using the NCBI Prokaryotic Genomes Automatic Annotation Pipeline (34). The G+C contents ranged from 33 to 39%, and between 1,922 and 2,520 genes were annotated per genome (Table 1).

**TABLE 1:** Genome characteristics and accession numbers of eight rodent helicobacters

Strain	GenBank accession no.	Host	Fold coverage	G+C content (%)	Estimated genome length (bp) using Velvet	No. of contigs using PGAP	No. of genes using PGAP
*H. muridarum* ST1	JRPD00000000	Mouse	56	33	2,354,445	92	2,351
*H. trogontum* (“*Flexispira rappini* taxon 6”) ATCC 700114	JRPL00000000	Rat	48	34	2,762,714	129	1,922
*H. typhlonius* MIT strain 97-6810	JRPF00000000	Mouse	62	38.5	1,899,179	25	2,520
*Helicobacter* sp. MIT strain 01-6451	JRMQ00000000	Mouse	89	37.5	2,056,937	48	2,064
*Helicobacter* sp. MIT strain 03-1614	JRMS00000000	Mouse	36	37.5	1,927,676	172	2,057
*Helicobacter* sp. MIT strain 03-1616	JROY00000000	Mouse	37	39	1,890,582	176	1,974
*Helicobacter* sp. MIT strain 05-5293	JROZ00000000	Wild mouse	65	38	2,016,563	101	2,097
*Helicobacter* sp. MIT strain 11-5569	JRPB00000000	Mouse	80	35	2,024,356	83	2,135

Due to the ability of EHS to interfere with biomedical research involving rodents, we evaluated the presence of known *Helicobacter* virulence determinants, such as gamma-glutamyl transpeptidase (*ggt*), cytolethal distending toxin subunit B (*cdtB*), and components of both the type IV and type VI secretion systems. Both *H. muridarum* and *H. trogontum* ATCC 700144 possess *ggt*, a *Helicobacter pylori* virulence factor that leads to cell cycle arrest, necrosis, and apoptosis (35). *cdtB* is present in *H. muridarum*, *H. typhlonius*, and the unnamed MIT strains 01-6451, 03-1614, 03-1616, and 05-5293. The entire *cdtABC* cluster was found in *H. muridarum* and the unnamed MIT strains 01-6451, 03-1614, and 05-5293. Multiple type IV secretion genes (*virB2-virB11* or *virD4*) were found in all species presented, excluding *H. muridarum* and MIT strain 01-6451. Type VI genes (*hcp*, *icmF, vasD*, and *vgrG*), associated with pathogenicity (36, 37), were less common. *icmF*, *vasD*, and *vgrG* were found in *H. trogontum* ATCC 700114 and the unnamed species MIT strain 03-1614. *vgrG* was found in *H. typhlonius* and several unnamed species (01-6451, 03-1616, and 11-5569).

### Nucleotide sequence accession numbers.

The genome sequences have been submitted to GenBank under the accession numbers listed in Table 1.
